# Fracture heat map of the facial skull demonstrates a danger zone of concomitant cervical spine injuries

**DOI:** 10.1038/s41598-021-91543-2

**Published:** 2021-06-07

**Authors:** Ákos Bicsák, Robert Sarge, Oliver Müller, Stefan Hassfeld, Lars Bonitz

**Affiliations:** 1 Department of Oral- and Maxillofacial Surgery, Facial Plastic Surgery, Dortmund General Hospital, Muensterstrasse 240, 44145 Dortmund, Germany; 2 Department of the University of Witten-Herdecke, Alfred-Herrhausen-Strasse 45, 58453 Witten, Germany; 3 Department of Neurosurgery, Dortmund General Hospital, Muensterstrasse 240, 44145 Dortmund, Germany

**Keywords:** Health occupations, Medical research, Neurology, Signs and symptoms

## Abstract

Concomitant maxillofacial and cervical spine injuries occur in 0.8–12% of the cases. We examined the relation of injury localization and the probability of cervical spine fracture. A retrospective study was conducted on patients that have been treated at Dortmund General Hospital for injuries both to the maxillofacial region and to the cervical spine between January 1st, 2007 and December 31th, 2017. Descriptive statistical methods were used to describe the correlation of cervical spine injuries with gender, age as well as maxillofacial injury localization. 7708 patients were hospitalized with maxillofacial injury, among them 173 were identified with cervical spine injury. The average ages for both genders lie remarkably above the average of all maxillofacial trauma patients (36.2 y.o. in male and 50.9 y.o. in female). In the group of men, most injuries were found between the ages of 50 and 65. Whereas most injuries among women occurred after the age of 80. The relative ratio of cervical spine injuries (CSI) varies between 1.1 and 5.26% of the maxillofacial injuries (MFI), being highest in the soft tissue injury group, patients with forehead fractures (3.12%) and patients with panfacial fractures (2.52%). Further, nasal, Le Fort I and II, zygomatic complex and mandibular condyle fractures are often associated with CSI. Fractures next to the Frankfurt horizontal plane represent 87.7% of all MFI with concomitant CSI. Patients in critical age groups with a high-energy injury are more likely to suffer both, MFI and CSI injuries. Our findings help to avoid missing the diagnosis of cervical spine injury in maxillofacial trauma patients.

## Introduction

Both maxillofacial injuries (MFI) and cervical spine injuries (CSI) are well-known to maxillofacial surgeons and neurosurgeon treating trauma cases. Nevertheless, only a few papers are handling concomitant MFI and CSI (MFI–CSI). The incidence of MFI–CSI ranges from 0.8 to 12% according to the recent literature^1–6^. However, the largest registry studies, report an incidence of 1.1–11.3%^7–10^, single-center studies estimate the occurrence of 0.8–9.7%^1–3,11^. In lethally injured patients this incidence is reported as high as 46.4%^12^.

The injuries of the maxillofacial region (MFI) in these patients include soft tissue injuries as well as complex panfacial fractures^10,13–15^. The injury pattern of the cervical spine (CSI) ranges from distortion and severe bony or ligamentous injuries to injuries of the spinal cord itself^11,15–19^. An inadequate or delayed diagnosis can have disastrous consequences and may even lead to the death of the patient. It seems that fatal cases are more common in combined MFI–CSI^12^. Still, CSI is often misdiagnosed even though the awareness of a thoroughly examination of the patients should be in routine practice of every trauma center^18,20–23^. Since severe cervical spine injuries can also be asymptomatic, it is important to perform an emergency radiologic assessment, for this purpose according to the ATLS guidelines^9,18,20,21,23–25^. A proper initial assessment of the patient at the accident site and in the emergency room allows the initiation of adequate therapy, and if required, surgical therapy for both MFI and CSI to decrease overall complications^5,21,25^.

Thorough knowledge of the relation of facial injuries and cervical spine injuries helps with initial patient assessment even in stressful situations and can avoid delayed or missed diagnosis with potentially catastrophic consequences. This study aims to analyze the data from the Dortmund Maxillofacial Trauma Registry from the years 2007 to 2017 and to compare these data with the international literature. The most important research question of the study is which patients are mostly endangered to suffer MFI–CSI and whether there is a specific fracture site localization of the maxillofacial region that predisposes to cervical spine injuries?

## Methods

The Ethics Committee of the University of Witten-Herdecke granted a written exemption for this retrospective study (152/2017).

We conducted a retrospective analysis of the patient data accessed in the hospital electronic repository. The analysis was carried out by selecting the appropriate patients using the codes of the disease-related groups (DRG). All patient records that have been treated at our hospital for injuries both to the head and neck region and to the cervical spine between 1st of January and 31th of December. 2017 were analyzed for study purposes.

Our Trauma Center is the highest level regional center also responsible for polytrauma care. Upon patient entry at the interdisciplinary Emergency Room, after an initial assessment, if a head and neck injury is obvious or suspected or the patient suffered a high-energy trauma the trauma mechanism is unknown or highly dangerous for a neurotrauma or spinal trauma, a high-resolution spiral computed tomography scan of the skull, face and cervical spine is routinely performed and by both radiologist and attending traumatologist, neurosurgeon and maxillofacial surgeon. If an indication for a magnetic resonance imaging is given, this is also immediately performed. Patients with neurotrauma are observed and regularly assessed in our Intensive Care Unit, if a follow-up radiology is required, this is also performed.

The fracture sites of the facial skull were analyzed by an experienced maxillofacial surgeon. Soft tissue injury data was collected based on the electronic document repository. The injuries to the cervical spine were analyzed by an experienced neurosurgeon. The demographic data regarding the population of the City of Dortmund have been acquired from the official demographic record published by the City Council Department. The statistical analysis was performed using descriptive statistical methods with Microsoft Excel 2013 (Microsoft Corp., Redmond, US). The epidemiological data were standardized based on the general demographic data (age and gender distribution) presented by the Authorities of the City of Dortmund^26^. The analysis of statistical significance was performed with standard chi-square (χ^2^)-test.

### Ethics committee approval, legal information

The Ethics Commission of the University of Witten—Herdecke has approved this study (No. 152/2017). The study was conducted in concordance with the Helsinki Declaration, ICH-GCP, laws and regulations of the European Union, those of the Federal Republic of Germany and of the State North-Rhine-Westphalia and with the internal regulations of Dortmund General Hospital. All included patients have signed an informed consent form.

## Results

In the study period from January 2007 to December 2017 a total of 7008 patients have been treated with maxillofacial injuries (Table 1). Among them, 4790 were male and 2218 female. A total of 173 patients had CSI, among them 106 male and 67 females. The gender difference is both in the total study population and in patients with CSI significant (χ^2^-test). The minimum age was 3.2 years, the oldest patient was 95 years old, both female. In the male the youngest patient was 3.5 y.o., the oldest one was 93.3 y.o.

**Table 1:** Demographics of the study population. ^a^Refers to age data in the patient group with concomitant CSI. p-values represent the p-values of χ^2^-test analyzing the difference between the two genders (confidence interval: p = 0.05).

	Male	Female	Total	p-value
Total number of patients with MFI	4790	2218	7008	**< 0.001**
Patients with CSI	106	67	173	**< 0.001**
Mean age^a^	49.2	55.8	52.3	–
Minimum age^a^	3.5	3.2	3.2	–
Maximum age^a^	93.3	95	95	–

**Figure 1:**
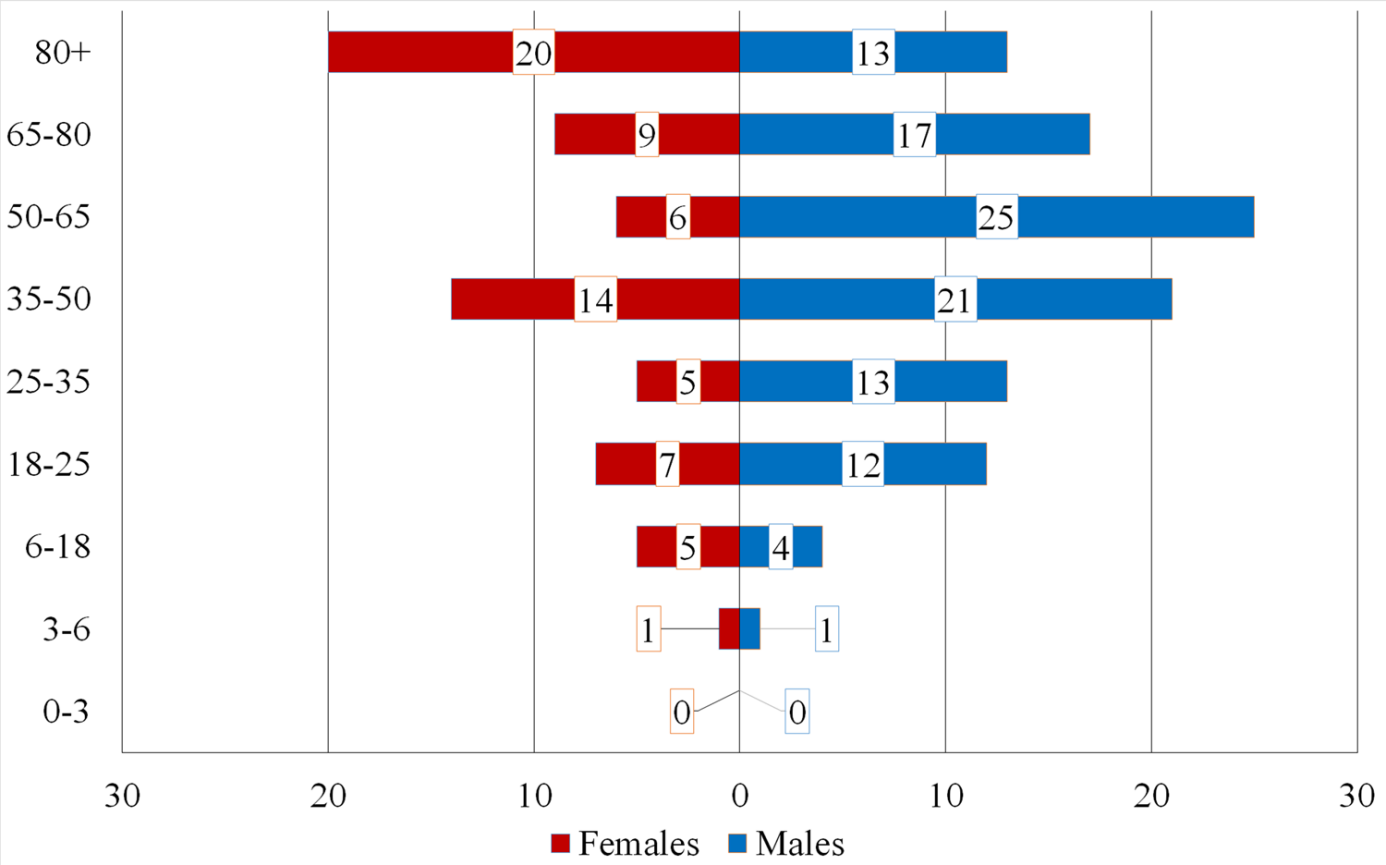
Histogram of the demographics of patients with concomitant CSI and MSI.

**Figure 2:**
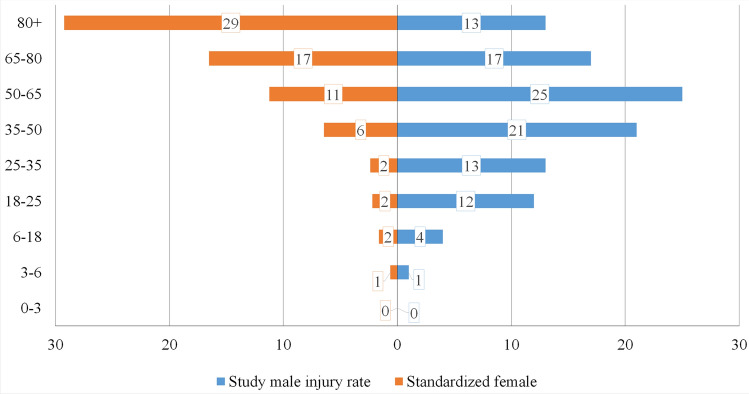
Standardized histogram based on the population registry report released by the Authority of the City of Dortmund of the patients with concomitant CSI and MSI. The age grouping in Figs. 1 and 2 is based on the official demographic registry report for comparability.

**Figure 3:**
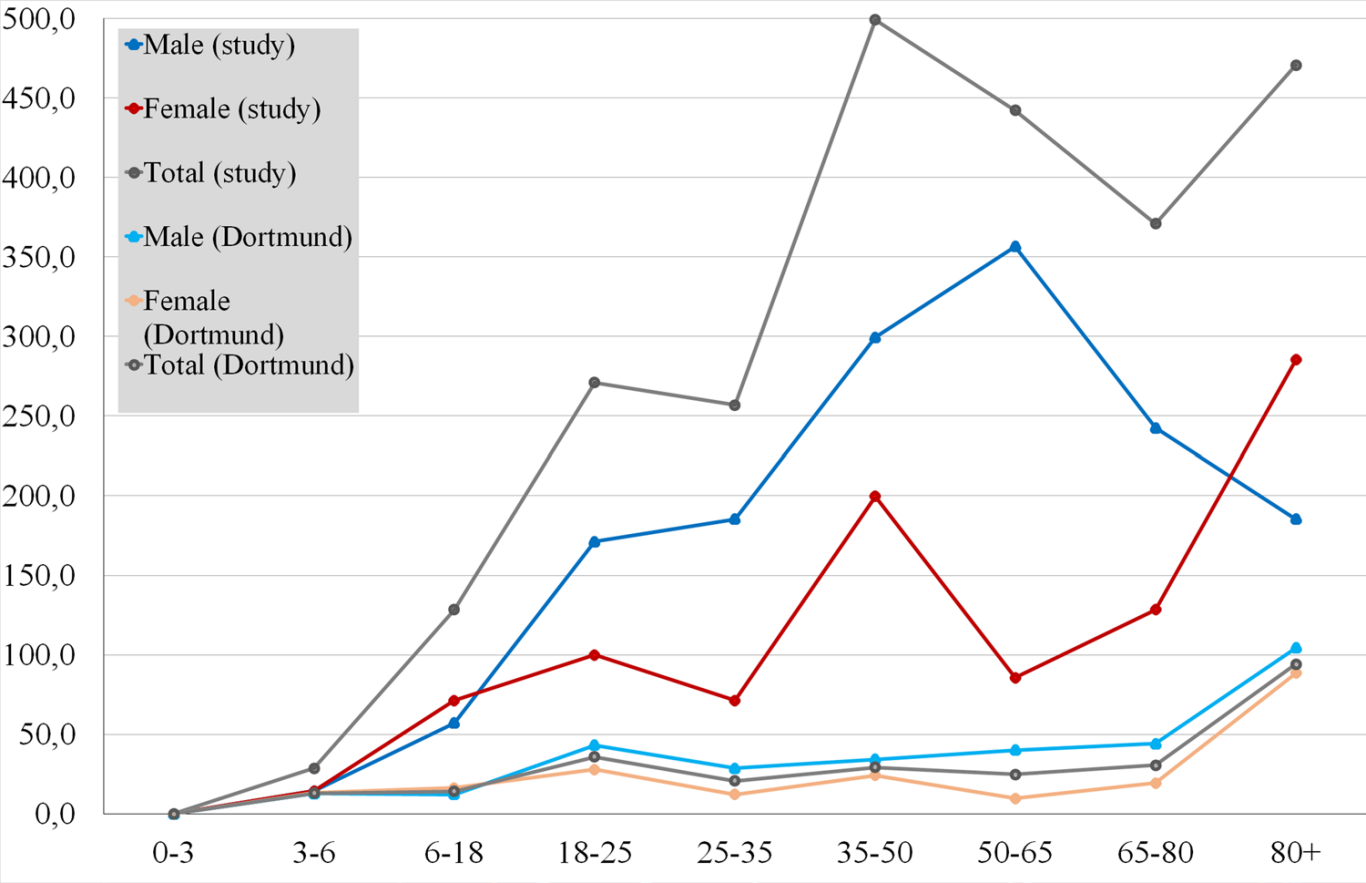
The incidence of CSI in the study population and MFI–CSI incidence in the whole population of Dortmund (1/100.000 inhabitants).

**Figure 4:**
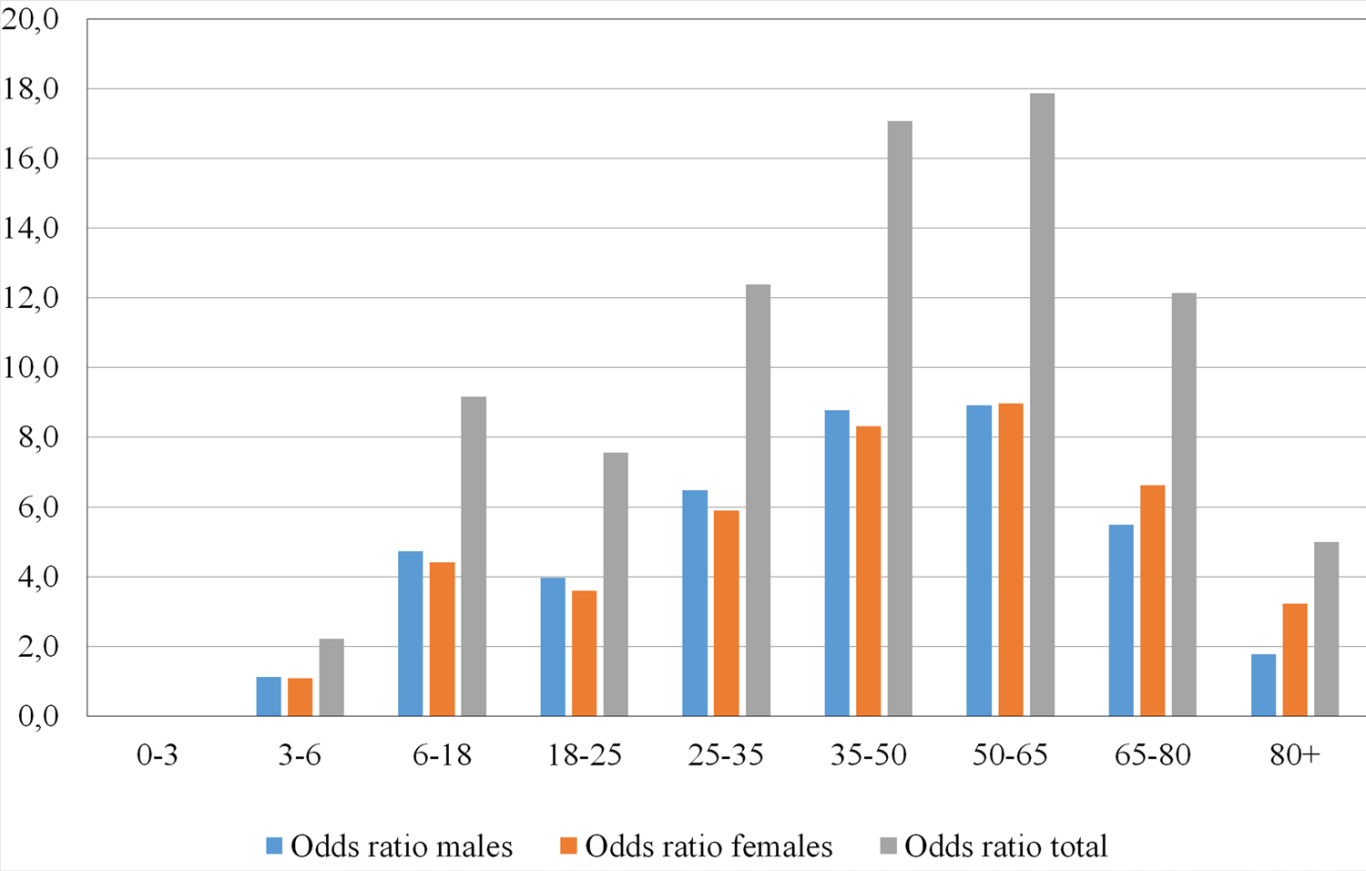
Presentation of the odds ratio in different gender and age groups.

Figure 1 shows the raw age-related distribution of MFI–CSI patients. The statistical analysis was based on the official demographic report of the City of Dortmund^26^. In the group of men, most injuries were found between the ages of 50 and 65, while females have the highest risk above 80 years of age. The standardized histogram (Fig. 2) and the average age of injured (male 49.2 y.o., female 55.8 y.o.) in Table 1 confirm these high-risk ages. The average ages for both genders lie remarkably above the average ages of all patients with MFI in our registry (36.2 y.o. in male and 50.9 y.o. in female). Due to this significant difference between both genders, a standardization of the age-related data was performed to reduce bias originating from the differently sized groups. The difference in males is higher than in the female. After standardization of the data (Fig. 1) is this effect clearly visible (Fig. 2). Male patients have a peak of risk at the age of 50–65 y.o., their risk decreases in the highest age groups. Figure 3 shows the age-related incidence of CSI in MFI patients and in relation to the city population. The odds ratio of acquiring a CSI in an MFI patient is presented in Fig. 4. The analysis of this diagram also confirms the higher risk of females in older age.

Table 2 shows the distribution of patients based on the MFI levels and the rate of CSI in each patient group. This grouping includes all types of injuries. The difference in each group is statistically significant (χ^2^-test, confidence interval: p < 0.05), except for the soft tissue injuries, where a trend is to be seen. The relative ratio of CSI varies between 1.1 and 5.26% and is high in the soft tissue injury group, in correlation with the injuries of the upper face and in patients with panfacial fractures.

**Table 2:** Patient distribution with different MFI with reference to the absolute numbers and ratio of CSI in each group. Significant p-values are typed in bold (χ^2^-test, confidence interval: p < 0.05). P-values represent the p-values of χ^2^-test analyzing the difference between the two groups (confidence interval: p = 0.05).

	CSI not detected	CSI present	Total	%-CSI	p-value
Soft tissue only	1441	80	1521	5.26%	0.162
Upper face	404	13	417	3.12%	**< 0.001**
Central midface	1477	27	1504	1.80%	**< 0.001**
Lateral midface	1348	15	1363	1.10%	**< 0.001**
Mandible	1077	12	1089	1.10%	**< 0.001**
Panfacial fracture	888	23	911	2.52%	**< 0.001**
Dentoalveolar only	191	3	194	1.55%	**< 0.001**

Table 3 presents the relation of the MFI and different CSI diagnoses. On average, 2.47% of all patients with MFI presented with CSI. Most injuries to the CS are distorsions (139, 80% of all CSI). Fractures were identified in 20 cases (11.6% of all CSI) and traumatic stenosis of the spinal canal was diagnosed in 7 cases (4% of all CSI). All these cases were due to disrupture or dislocation of the disc or dislocated bony fragments. No listhesis was found.

**Table 3:** The relation between the level of MFI and the detailed CSI diagnosis. *CS* cervical spine. The numbers represent absolute values.

MFI level	Total number of patients	Distorsion of the CS	Fracture CS	CS ligament rupture	Traumatic stenosis of the spinal canal	Hematoma	Stenosis of the spinal canal	Dura injury	Contusion of the spinal cord
Soft tissue	80	78	1	0	1	0	0	0	0
Forehead	13	7	4	1	1	0	0	0	0
Central midface	27	22	5	0	0	1	0	1	0
Lateral midface	15	11	2	1	1	0	0	0	0
Mandible	12	7	2	1	2	0	0	0	0
Polytrauma	23	11	6	0	2	0	2	0	2
Dentoalveolar injuries	3	3	0	0	0	0	0	0	0
Total	173	139	20	3	7	1	2	1	2

**Figure 5:**
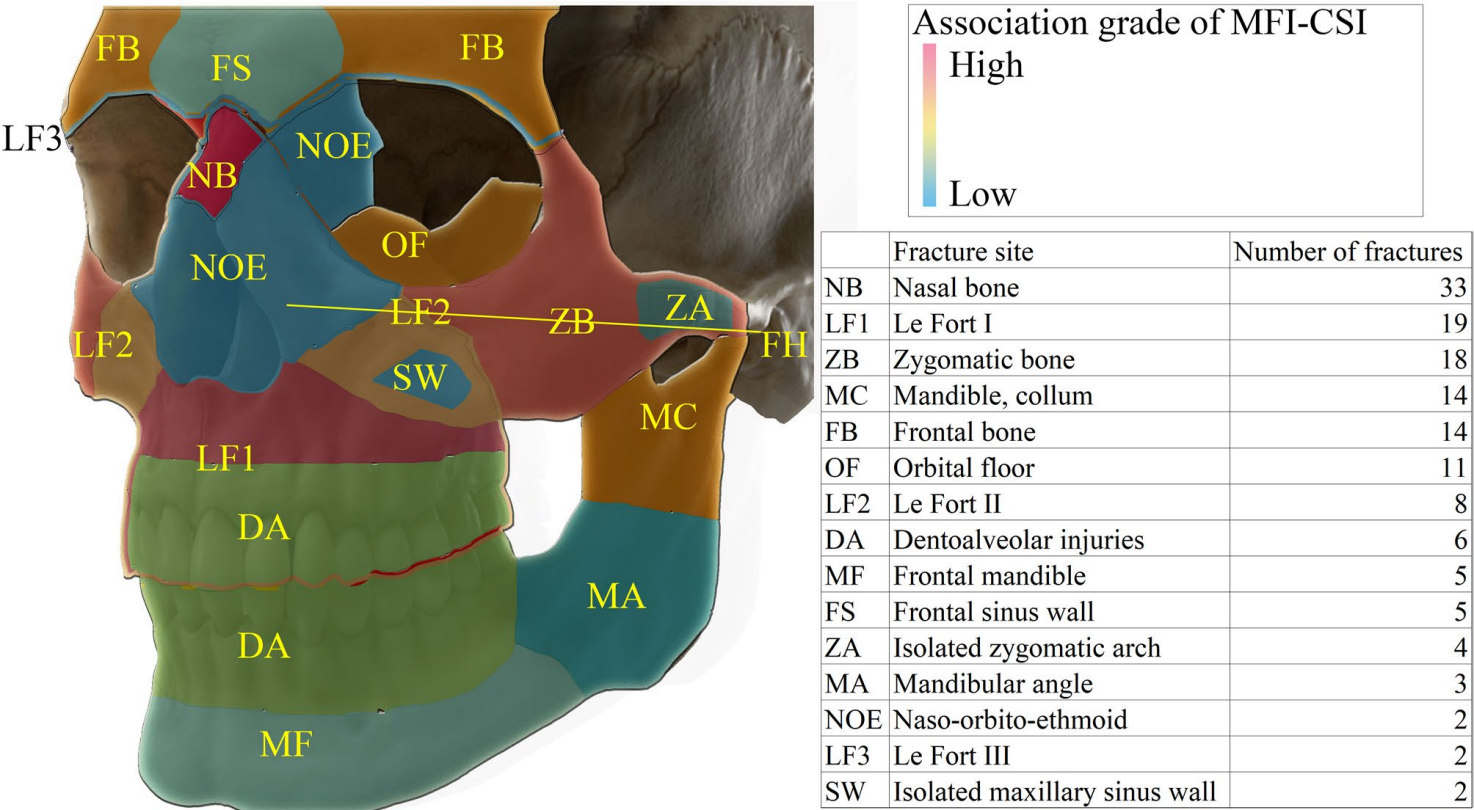
Heat map showing the areas of the skull with a higher risk of CSI. Fractures of the forehead, the nose, Le Fort I and II-level, zygomatic complex and the mandibular condyle are more often associated with CSI. The table includes the total number of fracture sites in MFI–CSI patients and thus does not refer to the number of patients. The yellow line represents the Frankfurt horizontal line on the left side.

In Fig. 5 we present the visualization of the most common fracture sites in the facial skull that are associated with CSI. These are located near to the midline, like the (central) forehead, nose and LeFort I and II level or in the lateral area of the face (zygomatic bone, orbital floor or mandibular condyle). In comparison, the injuries of the mandibular angle or front, the dentoalveolar or nasoethmoidal complex represent a lower risk of CSI. This heat map provides a good visualization that fractures of the facial skull near to the Frankfurt-horizontal plane are mostly connected with CSI. In this zone, we observed 87.7% of all fractures (128 of 146). This difference is statistically highly significant (p < 0.00001, *χ*^2^*-test, confidence interval: p* < 0.05).

As per our retrospective assessment, in 17 (10%) of all cases was the CSI delayed diagnosed. A completely missing diagnosis was not verified.

## Discussion

The initial assessment of patients in the emergency room is essential to detect life-threatening injuries or injuries that can lead to permanent disabilities^20–22,25^. Most maxillofacial injuries can easily be diagnosed, as the symptoms are relatively easy to assess. Nevertheless, severe injuries of the cervical spine can occur without alarming signs. Especially in unconscious patients, a possible injury to the cervical spine must always be considered both at the scene of the accident and in the emergency room. Many authors point out that injuries of the CS are often misdiagnosed^16,23^.

The highest incidence of concomitant MSI–CSI was detected in older patients than center average of maxillofacial trauma patients. In our study, 2.47% of all MFI patients were diagnosed with a concomitant CSI. This is comparable with the literature but is lower than the international average (0.8%^3^ to 12%^5^). This result is less than reported from the German registry study by Pietzka et al. (11.3%) or single-center studies from the UK or USA^1,2,11^, but more than 0.8% reported by Roccia et al. from Italy^3^. All these studies analyze a similarly long period of time, Färkkilä et al. from the same years^1,2^.

Our study presents a clear demographic trend. In female patients with MFI, there is an increasing risk with increasing age to suffer a concomitant CSI.

We found that injuries located near to the cranial base (most common fracture sites were the forehead, nose, LeFort I and II level, zygomatic bone, orbital floor or mandibular condyle) both in central and in lateral areas of the facial skull are significantly more often presented with CSI than other areas of the face (87.7% of cases with CSI). This level represents approximately the plane of the Frankfurt horizontal. Fractures in this area may point to a far more dangerous injury of the spine, thus from our data we suggest this region can be referred to as the “Cervical Spine Injury Alert Bend” of the face. To us, it is an important finding that soft tissue injuries of the face even without jaw fractures are associated with CSI as high as 5.25% range of all soft tissue injuries (p = 0.162). The soft tissue injury sites were often less precisely documented; thus, a mapping was not possible. Based on the findings in the fracture group, we suppose that the same distribution will apply for patients that suffered soft tissue injuries only. In contradiction to our study, Färkkilä et al. report in total higher MFI–CSI rates in patients with mandibular fractures than in those with midface fractures^1,2^. After a detailed analysis of these papers, a higher incidence of CSI is reported in more cranial fractures of the lower jaw, like fractures of the mandibular collum^2^. Their analysis of concomitant midfacial fractures and CSI provided a very similar result^1^ than our study.

In 17 cases (10%) of all CSI-MFI cases were delayed diagnosis. In these cases the CSI was detected in the post-injury surveillance phase. There were no missing diagnoses or re-admissions due to a missing CSI diagnosis. This amount of delayed diagnosis can be decreased by adequate guidelines, better initial assessment and staff training. Therefore, it is advisable to update both guidelines and center protocol and an immediate CT-scan of the skull, face and cervical spine should be performed in case of high energy accidents, traffic, falls from height, alterations of consciousness or injuries in the demonstrated danger zone even in case of minor trauma, especially in elderly^27^.

The above findings provide important guidance for the initial assessment in the emergency room, too. We suggest considering a high-resolution CT-scan of both the cervical spine (C1–C7) and the complete facial skeleton, if

1. there is an injury in the above-described zone, and
2. the patient is a male 35–65 years old or a female above 60 years of age, and
3. the injury mechanism suggests a middle to the high-energy impact of the head with fronto-posterior hyperextension of cervical spine^12^ or high shear forces in the lateral midface, or
4. Any high-energy trauma.

These clinical findings reflect the statements of Tuchtan et al. resulting from the finite element analysis of the projection of von Mises-forces after facial blunt trauma^28^. This paper reports that high antero-posterior forces can result in injuries to the ligaments, blood vessels, spinal cord or brain stem. Živković et al. report the same injuries in autopsy reports^12^. To the best of our knowledge, this is the first report that correlates MFI to CSI based on a large clinical data analysis. Our findings correlate to both virtual modeling and postmortem studies.

From our data, we strongly suggest that a thorough patient examination should be conducted by both, experienced maxillofacial surgeons and neurosurgeons, in critically injured patients presenting with the “Facial Alert Band” (FAB). This may help to avoid diagnostic failures or delayed diagnosis, especially in unconscious patients^23^.

In conclusion, injuries to the cervical spine in patients with maxillofacial injuries can be life-threatening or can cause severe life-long disability. The findings and the heat map presented in this paper can be a useful clinical tool even for an experienced team. It can reduce missing or delayed diagnosis of CSI, thus it helping to reduce possible complications, improve treatment outcome and quality and avoid legal consequences.
